# Research on the Sound Insulation Performance of Composite Rubber Reinforced with Hollow Glass Microsphere Based on Acoustic Finite Element Simulation

**DOI:** 10.3390/polym15030611

**Published:** 2023-01-25

**Authors:** Xiaocui Yang, Shuai Tang, Xinmin Shen, Wenqiang Peng

**Affiliations:** 1 Engineering Training Center, Nanjing Vocational University of Industry Technology, Nanjing 210023, China; 2 MIIT Key Laboratory of Multifunctional Lightweight Materials and Structures (MLMS), Nanjing University of Aeronautics and Astronautics, Nanjing 210016, China; 3 Systems Engineering Institute, Academy of Military Sciences, Beijing 100071, China; 4 Field Engineering College, Army Engineering University of PLA, Nanjing 210007, China; 5 College of Aerospace Science and Engineering, National University of Defense Technology, Changsha 410073, China

**Keywords:** composite rubber, hollow glass microsphere, acoustic finite element simulation, sound insulation mechanism, sound transmission loss, sound pressure level

## Abstract

The composite rubber reinforced with hollow glass microsphere (HGM) was a promising composite material for noise reduction, and its sound insulation mechanism was studied based on an acoustic finite element simulation to gain the appropriate parameter with certain constraint conditions. The built simulation model included the air domain, polymer domain and inorganic particles domain. The sound insulation mechanism of the composite material was investigated through distributions of the sound pressure and sound pressure level. The influences of the parameters on the sound transmission loss (STL) were researched one by one, such as the densities of the composite rubber and HGM, the acoustic velocities in the polymer and inorganic particle, the frequency of the incident wave, the thickness of the sound insulator, and the diameter, volume ratio and hollow ratio of the HGM. The weighted STL with the 1/3 octave band was treated as the evaluation criterion to compare the sound insulation property with the various parameters. For the limited thicknesses of 1 mm, 2 mm, 3 mm and 4 mm, the corresponding optimal weighted STL of the composite material reached 14.02 dB, 19.88 dB, 22.838 dB and 25.27 dB with the selected parameters, which exhibited an excellent sound insulation performance and could promote the practical applications of the proposed composite rubber reinforced with HGM.

## 1. Introduction

The characteristics of viscoelasticity and inner damping for rubber make it a promising material for noise reduction [1], which can obtain excellent sound insulation and vibration attenuation, simultaneously [2]. Meanwhile, it has the advantages of possessing a light weight, fine machinability, outstanding physical and mechanical performances, a small occurred space, can facilitate construction, and so on [3,4]. Thus, rubber products have been widely utilized for sound insulation in the fields of communications and in the transportation industry (such as high speed rail, massive ships, passenger planes and vehicles), the construction industry, electrical equipment, industrial buildings, hospitals, educational institutions, guesthouses, and so on [5,6,7,8]. This makes it a focus of research in the domain of polymeric material and has attracted enthusiastic research interests all over the world. For example, the crumb rubber asphalt mixtures had been utilized to gain newly laid low-noise pavements [9,10,11,12], which could reduce the generated noise in urban traffic.

In order to further improve the sound insulation property of the rubber products, some functional reinforced fillers are added to develop the composite rubber by the mechanical or solution blending methods [13,14], and the normal utilized fillers include the metallic hollow sphere [15], ultrafine metal powder [16], kenaf and calcium carbonate [17], micro CaCO_3_ and hollow glass microspheres (HGM) [18], Eichhornia crassipes fiber (ECF) and maleates of Eichhornia crassipes fiber (MoECF) [19], recycled fir sawdust [20], etc. The polyurethane/316L stainless steel hollow spheres and silicone rubber/316L stainless steel hollow sphere composites had been prepared by Yu et al. [15] using the casting method, which proved that the different proportions of free volume in the polyurethane and silicone rubber matrix was a major reason for the significant differences in their sound absorption properties. Hu et al. [16] proved that the microcellular foaming material exhibited the best performance of sound insulation with the superfine metal powder content in matrix 30 wt%. The rubber composites as sound insulators were prepared by Suhawati et al. [17] through the incorporation of two types of fillers, namely kenaf and calcium carbonate, in blends of 50 mole% epoxidized natural rubber and methyl methacrylate–grafted natural rubber latex. It had been proved by Fang et al. [18] that, compared to the pure polymer sample, both the CaCO_3_ and HGM–filled thermoplastic elastomer composites exhibited greatly enhanced the soundproofing efficiency, which increased the sound transmission loss (STL) value from the original 29 dB to 45 dB. El–Wakil et al. [19] proved that the styrene–butadiene rubber composite with 10 phr of MoECF had sound absorption amplitude equal to 0.9 at the frequency of 400 Hz, and the sound absorption performance improved in low–frequency regions below 500 Hz by increasing the thickness to 2.3 mm. The mixing rigid polyurethane foam (RPUF)/flexible polyurethane foam (FPUF) with 0, 35, 40, 45, and 50 wt% fir sawdust was prepared by Tiuc et al. [20], and the obtained composite materials containing 50% sawdust had superior acoustic properties compared to those with 100% FPUF in the frequency range of 420–1250 Hz. Meanwhile, graphene nanoplatelet [21], organoclay montmorillonite [21], glass fiber [22], magnetite and barite minerals [23] have also been utilized as functional reinforced fillers, all of which aim to further improve the sound insulation performance of the rubber products.

Among these functional reinforced fillers [15,16,17,18,19,20,21,22,23], the HGM is a novel hollow spherical particle, which has the advantages of light weight, low density, excellent heat insulation performance, high pressure resistance, outstanding fire–resistant insulation property, and the fine sound insulation capacity, which is considered as a potential functional material for the fields of construction, transportation, machinery, aerospace, military, etc. [24,25,26,27,28,29,30]. Du and He [24] reviewed the progress made in synthesis and applications of the spherical silica micro/nanomaterials with multilevel (hierarchical) structures, which might enable them to be used in the broad and promising applications as ideal scaffolds (carriers) for biological, medical, and catalytic applications. An overview of the fabrication techniques of bulk and hollow microspheres was provided by Righini [25], as well as of the excellent results made possible by the peculiar properties of microspheres. To take advantage of both the low density and thermal conductivity of HGM, and the high mechanical and electrical conductivity of the carbon–based nanofiller, micro– and nanosized filler were combined into a single composite material by Herrera–Ramirez et al. [26]. An and Zhang [27] fabricated the core/shell structured glass/Ni–P/Co–Fe–P composite hollow microspheres by a three–step route, which showed their promising applications in the fields of low–density magnetic materials, conduction, and catalysis, etc. Cho et al. [28] had modelled the elastoplastic deformation behaviors of HGM/iron syntactic foam under tension by using a representative volume element (RVE) approach, which exhibited the potential for applications in the design of composites with a high modulus matrix and high strength reinforcement. Composite foamy structures were prepared by An and Zhang [29] through the HGM-assisted bubbling of silicone rubber with ammonium hydrogen carbonate as a blowing agent, and the proposed composite foamy structures improved the heat insulation and sound absorbing properties.

In order to improve the research efficiency and reduce the experiment cost, the acoustic finite element simulation has been widely utilized in the field of sound insulation and noise reduction [30,31,32,33,34,35,36,37,38,39]. Okuzono et al. [30] applied the finite element method using hexahedral 27-node spline acoustic elements with low numerical dispersion for the room acoustics simulation in both the frequency and time domains. The combination of the finite element simulation and cuckoo search algorithm was utilized by Yang et al. [31] to optimize the sound absorption property of the acoustic metamaterial of multiple parallel hexagonal Helmholtz resonators with sub-wavelength dimensions, in which the simulation results and experimental results exhibited an excellent consistency. Sathyan et al. [32] proposed a numerical method combining both the finite element method and boundary element method for the acoustic noise of electromagnetic origin generated by an induction motor. In order to improve the research efficiency, Wang et al. [33] used a two–dimensional equivalent simulation model to obtain the initial value of the parameters and a three–dimensional finite element model to simulate the sound absorption performance of a metamaterial cell. The finite element analysis procedure was selected by Abdullahi and Oyadiji [34] to simulate wave propagation in air-filled pipes, which was essential in the study of wave propagation in pipe networks such as oil and gas pipelines and urban water distribution networks. Yang et al. [35] used the finite element method to exhibit the sound absorption mechanism of adjustable parallel Helmholtz acoustic metamaterial through the distribution of sound pressures for the peak absorption frequency points. Van Genechten et al. [36] developed a hybrid simulation technique for coupled structural-acoustic analysis, which included a wave-based model for acoustic cavity and a direct- or modally-reduced finite element model for the structural part. The influence of tunable aperture with a variable length was investigated by Yang et al. [37] through an acoustic finite element simulation with a two-dimensional rotational symmetric model, which were consistent with the experimental results. Lin et al. [38] used the finite element simulation method and the experiment testing to validate the sound insulation performances of a novel sandwich structure compounded with a resonant acoustic metamaterial. Acoustic finite element numerical simulation analysis of the sound insulation hood model was carried out using the acoustic software LMS Virtual Lab Acoustics by Wu et al. [39], and the simulation result was verified by the experimental validation. It has been proved by these literatures [30,31,32,33,34,35,36,37,38,39] that the acoustic finite element simulation is an effective and helpful method to analyze the sound characteristics of materials or structures through selecting the suitable mesh type and appropriate element parameters, which is propitious for improving research efficiency and reducing the experimental steps and costs.

Therefore, the sound insulation performance of the composite rubber reinforced with HGM was investigated by acoustic finite element simulation in this research, which aimed to promote its practical application in the field of noise reduction. Meanwhile, the weighted STL with the 1/3 octave band was treated as the evaluation criterion for comparing the sound insulation performance with various influencing parameters [40,41,42]. The finite element simulation model was first built based on the basic theory of pressure acoustics [43,44], which could research the sound insulation mechanism of the composite material through analyzing the distribution of the sound pressure level (SPL). Afterward, the influences of the parameters on the STL of the composite materials were investigated one by one, such as the density of the composite rubber and that of the HGM, the acoustic velocity in the polymer and that in the inorganic particle, the frequency of the incident wave, the thickness of the sound insulator, and the diameter, volume ratio and hollow ratio of the HGM. Later, based on the achieved effect behaviors of the influencing parameters, the weighted STL of the composite material for the limited thickness of the sound insulator was optimized through parameter optimization with the neural network algorithm [45,46,47,48], which aimed to obtain the optimal sound insulation effect with certain constraint conditions. The proposed sound insulation material of composite rubber reinforced with HGM could be considered as a highly efficient sound insulator with little occupied space, which could be favorable for promoting its practical application in the industrial field. In general, the object of this study is to improve the sound insulation performance of the composite rubber reinforced with HGM, and the major method is investigating the influencing principle of each parameter on the SPL based on the acoustic finite element simulation model. The major achievements gained in this research, on the influencing principle of each parameter and the exhibition of the sound insulation mechanism, would provide effective guidance and meaningful reference for the development of a novel sound insulator.

## 2. Acoustic Finite Element Simulation Model

The acoustic finite element simulation model was built based on the basic theory of pressure acoustics [43,44], and it supplied the foundation to investigate the sound insulation mechanism of the composite material by analyzing the distributions of the SPL.

### 2.1. Model Construction

**Figure 1:**
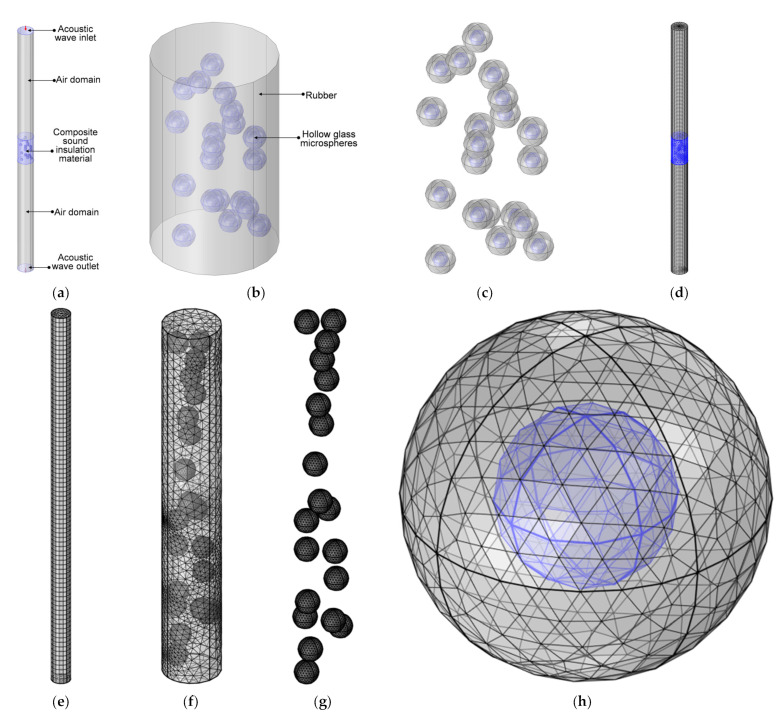
The constructed finite element simulation model. (**a**) General structure of whole model; (**b**) the sound insulator; (**c**) the HGMs; (**d**) the gridded model of whole finite element structure; (**e**) the gridded model of air domain; (**f**) the gridded model of sound insulator; (**g**) the gridded model of the HGMs; (**h**) the gridded model of single HGM.

The constructed acoustic finite element simulation model for the composite rubber reinforced with HGM based on the basic theory of pressure acoustics is shown in Figure 1. It consisted of an air domain, polymer domain and inorganic particles domain, as shown in Figure 1a. The incident wave with a pressure amplitude of 1 Pa was set in the acoustic wave inlet. The composite sound insulation material consisted of the basic material of rubber and the filler of HGMs, as shown in Figure 1b. The details of these filled HGMs are shown Figure 1c, and the blue part in each HGM was the air. After setting the geometric parameters (such as thickness of the sound insulator, diameter, volume ratio and hollow ratio of the HGM, etc.) and physical parameters (such as density of the composite rubber and that of the HGM, acoustic velocity in the polymer and that in the inorganic particle, etc.), the geometric model was further gridded, as shown in the Figure 1d. The mesh type for the finite element simulation model was the free tetrahedron mesh, which could give considerations to both the simulation accuracy and computational efficiency, and the size of the elements in it was determined by the tiniest unit within the whole finite element structure, which was the hollow air domain inside the HGM in in this study. Thus, the selected smallest unit size and largest unit size for the composite sound insulation material, as shown in Figure 1a, were determined by the diameter, D_m_, and the hollow ratio, μ_h_, of the HGM; the former was set as μ_h_ × D_m_/10 and the latter was set as μ_h_ × D_m_/100. The mesh for the air domains in the Figure 1a was obtained by sweeping with the 80 fixed units, as shown in the Figure 1e. Meanwhile, the gridded models of the sound insulator, HGMs and single HGM are shown in Figure 1f, Figure 1g and Figure 1h, respectively. With the exception of the acoustic wave inlet and the acoustic wave outlet, the other boundaries were set as the hard boundary condition. The acoustic wave with vibration mode *P*_n_ = 1 and mode wavenumber *k*_n_ = 2 × π/(*C*_0_/acpr.freq) was set in the acoustic wave inlet, and its value was defined as 1 Pa and its phase was 0 rad. The STL was selected to evaluate the sound insulation performance with 1/3 octave band (the investigated frequencies were 100 Hz, 125 Hz, 160 Hz, 200 Hz, 250 Hz, 315 Hz, 400 Hz, 500 Hz, 630 Hz, 800 Hz, 1000 Hz, 1250 Hz, 1600 Hz, 2000 Hz, 2500 Hz and 3150 Hz) in this research, which was conversed by calculating the transmission loss at the acoustic wave outlet relative to the standard input at the acoustic wave inlet.

With the exception that the density of air and the acoustic velocity in the air were kept at the constant of 1.21 Kg/m^3^ and 343 m/s, respectively, the other parameters were selected in a reasonable range, which are summarized in Table 1. The reference values for each parameter were chosen as the median, which was treated as the selected parameters for the investigation of the sound insulation mechanism and the analysis of the influencing parameters.

### 2.2. Sound Insulation Mechanism

**Figure 2:**
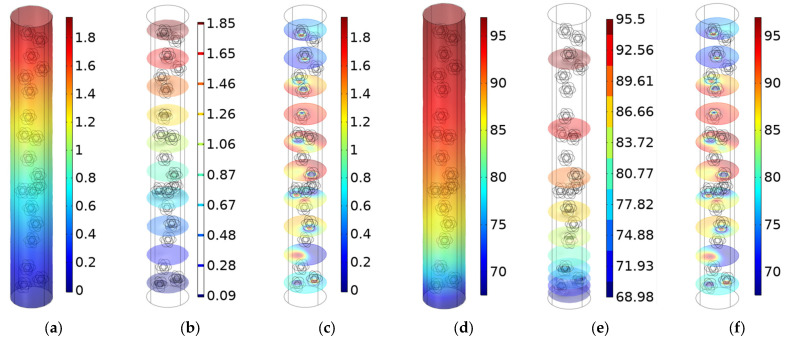
Sound insulation mechanism of the composite rubber reinforced with HGM. (**a**) Distribution of the SP; (**b**) the isosurfaces of the SP; (**c**) the sectional surfaces of the SP; (**d**) distribution of the SPL; (**e**) the isosurfaces of the SPL; (**f**) the sectional surfaces of the SPL.

The sound insulation mechanism of the composite rubber reinforced with the HGM was investigated through distributions of the sound pressure (SP) and those of the SPL, as shown in the Figure 2, which corresponded to the frequency of 1000 Hz for the composite rubber reinforced with HGM, selecting the parameters by the reference values in the Table 1.

It could be found that both the SP and SPL decreased along the thickness direction. The SP decreased from 1.8 to 0, judging from Figure 2a, and the SPL decreased from 95 dB to 70 dB, judging from Figure 2d. Meanwhile, it could be observed that the isosurfaces of the SP, shown in Figure 2b, were equally spaced from 1.85 to 0.09 with an approximate interval of 0.2, and the isosurfaces of the SP, in Figure 2b, were unequally spaced from 95.5 to 68.98 with the approximate interval of 2.95; this was consistent with the normal relationship between SP and SPL, as shown in Equation (1). Here, SP_ref_ is the reference sound pressure, which is 2 × 10^−5^ Pa for the propagation medium of air. Moreover, it could be found from the sectional surfaces of the SP, in Figure 2c, and those of the SPL, in Figure 2f, that the existence of the HGMs would significantly alter the sound wave propagation in the composite rubber. Then, the sound insulation mechanism was discussed based on the acoustic wave transmission process in the composite rubber reinforced with the HGM.

(1) $$\mathrm{SPL}=20\times \log_{10}\left(\frac{\mathrm{SP}}{{\mathrm{SP}}_{\mathrm{ref}}}\right)$$

**Figure 3:**
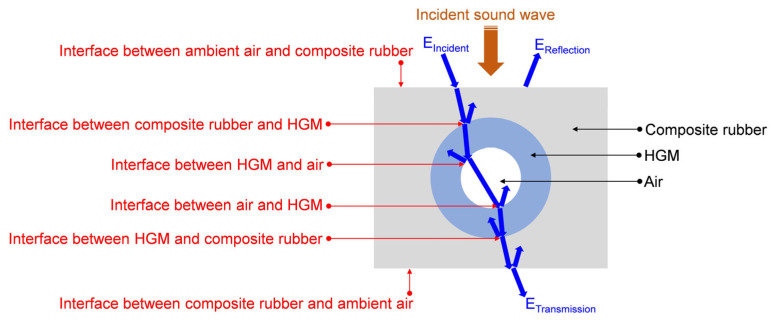
Schematic diagram of acoustic wave transmission process in the composite rubber reinforced with the HGM.

The schematic diagram of the acoustic wave transmission process in the composite rubber reinforced with the HGM is shown in the Figure 3. Among the composite rubbers, HGM and air (including the ambient air and the inside air in the HGM), there were six interfaces, as shown in the Figure 3. At each interface, there would be a reflection and transmission of the incident sound wave, which are exhibited by the blue arrows in Figure 3. When the incident sound energy E_Incident_ reached the interface between the ambient air and composite rubber, part of the sound energy was reflected back as E_Reflection_, and the other penetrated into the composite rubber. Similarly, there would be multiple reflections and refractions at the various interfaces, and the final transmission sound energy E_Transmission_ penetrated outside of the composite rubber. The STL could be calculated by Equation (2) for the condition of normal incidence. Here, P_i_ and P_t_ are the sound intensity of the incident wave and that of the transmission wave, respectively.

(2) $$\mathrm{STL}=10\log_{10}\left(\frac{P_{i}}{P_{t}}\right)$$

There were two major reasons to generate the sound insulation effect in the composite rubber reinforced with the HGM [49,50,51]. Firstly, there existed many interfaces with unmatched acoustic impedance among the air, composite rubber and HGM, as shown in Figure 3. These interfaces not only increased the reflection and diffraction of the sound wave, but could also extend the transmission path of the sound wave to consume more sound energy, which resulted in a decrease in the transmitted acoustic energy and an improvement in the sound insulation effect. Secondly, the hollow structures in the HGM could reflect the sound wave entering the cavity for many times to consume part of the sound energy, and the expansion and compression of the air in the cavity could translate the sound energy to kinetic energy and thermal energy of the air, which could further consume the sound energy to reduce the transmitted acoustic energy. It could be found that the sound insulation process in the composite rubber reinforced with the HGM was really complex, which indicated that the construction of the theoretical model based on the sound insulation mechanism was difficult to realize and the accuracy of the constructed model was limited. Thus, the acoustic finite element simulation method was selected to investigate the effects of the influencing parameters in this study, which could better simulate the actual acoustic wave transmission process in the proposed sound insulator of composite rubber reinforced with HGM.

## 3. Influencing Parameters

**Figure 4:**
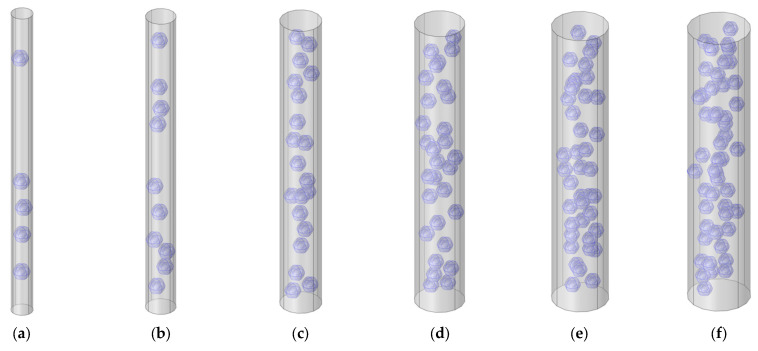
Acoustic finite element simulation models of the composite rubber reinforced with HGM for the various calculation quantity of the HGM. (**a**) 5; (**b**) 10; (**c**) 20; (**d**) 30; (**e**) 40; (**f**) 50.

The influencing parameters that affected the sound insulation effect of the composite rubber reinforced with the HGM could be divided into two groups. The first group was made up of the structural parameters, such as the diameter, volume ratio, and hollow ratio of the HGM and the thickness of the sound insulator. The second group was the physical parameters, such as the density of the composite rubber and HGM, and the acoustic velocity in the composite rubber and HGM. These eight influencing parameters were investigated one by one in this study. In the simulation process, the calculation quantity of the HGM was the major factor in determining the simulation accuracy. An increase in the calculation quantity could make the simulation process closer to the actual situation, which would lead to a higher simulation accuracy, but the simulation calculation amount would significantly increase, and the simulation time would remarkably extend. Thus, the establishment of the suitable calculation quantity of HGM should be confirmed first. The acoustic finite element simulation models of the composite rubber reinforced with HGM for the various calculation quantities of the HGM are shown in Figure 4, and the selected calculation quantities of the HGM were 5, 10, 20, 30, 40 and 50, respectively. The other parameters were the same as the reference values of the parameters in Table 1. The HGMs were randomly distributed in the rubber, which was realized by the random generation of the HGMs in the acoustic finite element simulation model. Supposing the diameter of the computational model was D_c_, and calculation quantity of the HGM was N, the volume of the HGM V_h_ and that of the whole sound insulator V_s_ could be calculated by Equations (3) and (4), respectively, according to the parameters listed in Table 1. Thus, according to the definition of the volume ratio of the HGM μ_v_, the value of D_c_ was confirmed by the Equation (5). That is why the D_c_ had become larger along with the increase in the calculation quantity of the HGM, as shown in Figure 4.

(3) Vh=N×43πDh/23=16NπDh3

(4) Vs=πDc/22×T=14πTDc2

(5) Dc=23NDh3/μv/T

The mesh partition is another pivotal factor influencing the simulation accuracy and efficiency. In order to give consideration to both the simulation efficiency and accuracy, the free tetrahedron mesh grid was utilized, and the minimum cell size was set as D_h_/20/μ_h,_ and the maximum cell size was set as D_h_/2/μ_h_ for the HGM domain; the minimum cell size was set as D_h_/20 and the maximum cell size was set as D_h_ for the composite rubber domain; the other domains were generated by sweeping with the distribution number of 80. These parameters for the mesh partition were applied to all of the finite element simulation models in this study.

**Figure 5:**
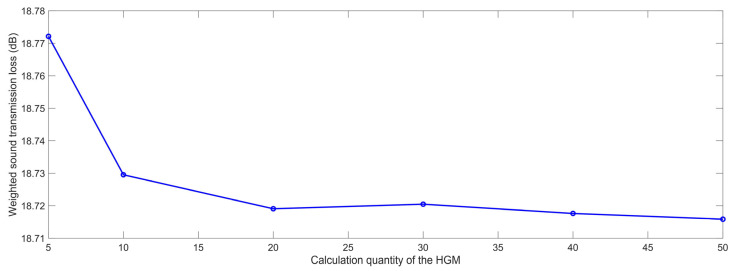
The weighted STL of composite rubber reinforced with HGM for the various calculation quantity of the HGM.

Based on the constructed acoustic finite element simulation model and the selected parameters, the STL data with the various calculation quantities of the HGM were gained, as shown in Table 2, and the change of the weighted STL along with the increase in the calculation quantity N is shown in Figure 5. It could be calculated that the undulation of the weighted STL was limited in 0.005 dB when the calculation quantity was larger than 20, which indicated that the calculation quantity 20 was enough to achieve accurate simulation results. Therefore, the calculation quantity N was selected as 20 for the following research in this study.

It was interesting to note that the weighted STL rose a little, from 18.7191 dB to 18.7205 dB, when the calculation quantity N increased from 20 to 30. The major reason for this phenomenon was that the generation of HGM in the composite rubber was completely random, and the uniformity of the distribution of the HGMs would affect the simulation accuracy. Normally, the uniformity would improve along with the increase in the calculation quantity N. However, for this particular simulation process, it could be judged that the uniformity of the distribution of the HGMs, when N = 20, as in Figure 4c, was better than that when N = 30, as in Figure 4d. Therefore, the uniformity of the distribution of the HGMs was taken into account in the following simulation process, and it would improve when the distribution of the HGMs in the composite rubber was as uniform as possible.

### 3.1. Structural Parameters

#### 3.1.1. Diameter of the HGM

**Figure 6:**
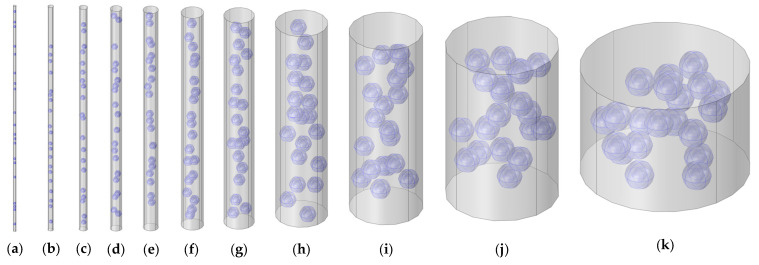
Acoustic finite element simulation models of the composite rubber reinforced with HGM for the various diameter of the HGM. (**a**) 20; (**b**) 30; (**c**) 40; (**d**) 50; (**e**) 60; (**f**) 80; (**g**) 100; (**h**) 150; (**i**) 200; (**j**) 300; (**k**) 500.

The acoustic finite element simulation models of the composite rubber reinforced with HGM for the various diameters of the HGM are shown in Figure 6, which select eleven samples in the value range of D_m_, and the other parameters select the reference values. It can be observed from Figure 6 that the diameter of the computational model D_c_ grew larger along with the increase in the D_m_, which was consistent with the calculation results for Equation (5). Based on the constructed acoustic finite element simulation models and the selected parameters, the STL data with the various diameters of the HGM were gained, as shown in Table 3. It could be found that the STL data at each investigated frequency point in the range of 100–3150 Hz decreased normally along with the increase in the diameter of the HGM, and this difference was more obvious for the high frequency region.

**Figure 7:**
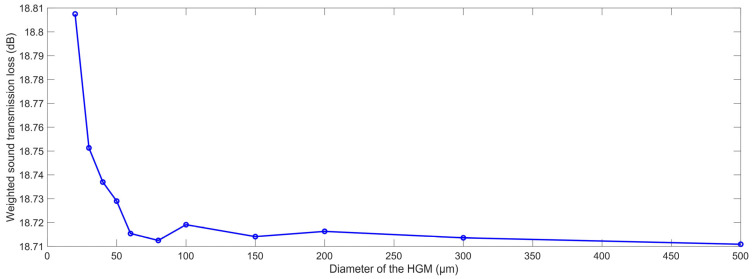
The weighted STL of composite rubber reinforced with HGM for the various diameter of the HGM.

The variation of the weighted STL along with the increase in the diameter of the HGM D_m_ is shown in the Figure 7. It could be found that the relationship between the weighted STL and the diameter of the HGM was negative, particularly when the value of the D_m_ was smaller than 60. The possible reason for this phenomenon was that the STL was proportional to the modulus of the elasticity of the material E, and the value of E decreased along with the increase in diameter of the HGM D_m_. Meanwhile, the HGM with a smaller diameter had the higher density, larger thickness of the wall and the higher rigidity, as shown in the acoustic wave transmission process in the composite rubber reinforced with the HGM in Figure 3, which generated more acoustic reflecting and diffractive interfaces to consume more acoustic energy. Moreover, the absolute value of the variation of the weighted STL was smaller than 0.1 dB with the increase in the D_m_ from 20 μm to 500 μm, and all of the weighted STL was maintained in range of 18.71–18.81 dB, which indicated that the relative change of the weighted STL was limited in 0.5%. The major reason for this phenomenon was that the volume of rubber was reduced along with the increase in the diameter of the HGM D_m_, because the volume ratio of the HGM was kept constant, which resulted in a smaller decrease in the equivalent modulus of the elasticity of the whole sound insulator and the STL was proportional to the modulus of the elasticity of the material E. However, the HGM with a larger diameter could result in more acoustic reflecting and diffractive interfaces, as shown in Figure 3, which would lead to an increase in the sound insulation effect. Thus, the final sound insulation performance was determined by the comprehensive effect. That is why the weighted STL decreased normally and there was a small increase in the weighted STL when the diameter of the HGM was 100 μm and 200 μm, respectively. Therefore, it could be concluded that the diameter of the HGM D_m_ had little impact on the sound insulation effect of the composite rubber reinforced with HGM, and its selection could pay more attention to the other factors, such as manufacturing cost, dispersion, uniformity, etc.

#### 3.1.2. Volume Ratio of the HGM

**Figure 8:**
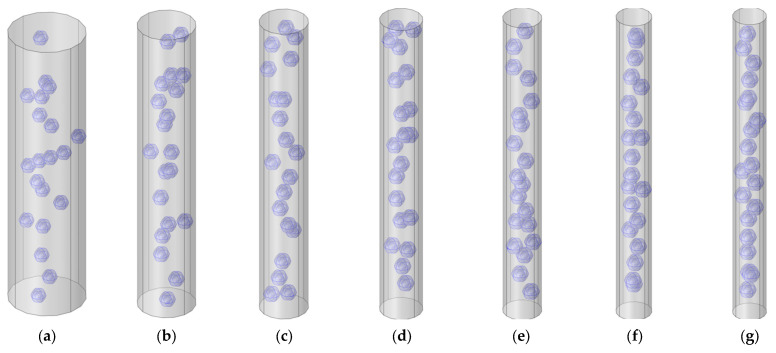
Acoustic finite element simulation models of composite rubber reinforced with HGM for various volume ratio of HGM. (**a**) 2.5%; (**b**) 5.0%; (**c**) 7.5%; (**d**) 10.0%; (**e**) 12.5%; (**f**) 15.0%; (**g**) 17.0%.

Similarly, the finite element simulation models of the composite rubber reinforced with HGM for various volume ratios of the HGM μ_v_ were built, as shown in Figure 8. When the μ_v_ is larger than 17.5%, the theoretical computational diameter D_c_ will be smaller than the diameter of the HGM D_m_, which indicated that the model cannot be constructed. Thus, the picked values for the μ_v_ were in the range of 2.5% to 17.0% in this research.

**Figure 9:**
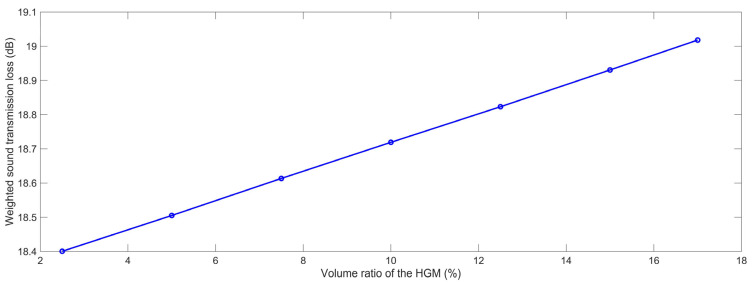
The weighted STL of composite rubber reinforced with HGM for the various volume ratio of the HGM.

Based on the constructed acoustic finite element simulation models and the selected parameters, the STL data with various volume ratios of the HGM were achieved, as shown in Table 4, and the variation of the weighted STL along with the increase in the volume ratio of the HGM μ_v_ is shown in Figure 9. It could be found that the relationship between the weighted STL and the volume ratio of the HGM μ_v_ was positive, which was almost linear. The major reason for this phenomenon was that more acoustic reflecting and diffractive interfaces were generated with the increase in the volume ratio of the HGM μ_v_, as shown in the acoustic wave transmission process in the composite rubber reinforced with the HGM in Figure 3, which could result in a greater consumption of the acoustic energy. However, the absolute value of the variation of the weighted STL was close to 0.6 dB, with the increase in the μ_v_ from 2.5% to 17%, because the increase in the volume ratio of the HGM μ_v_ occupied the space of the base material of the rubber, which indicated that the volume ratio of the the HGM also had little impact on the sound insulation effect of the composite rubber reinforced with HGM.

#### 3.1.3. Hollow Ratio of the HGM

**Figure 10:**
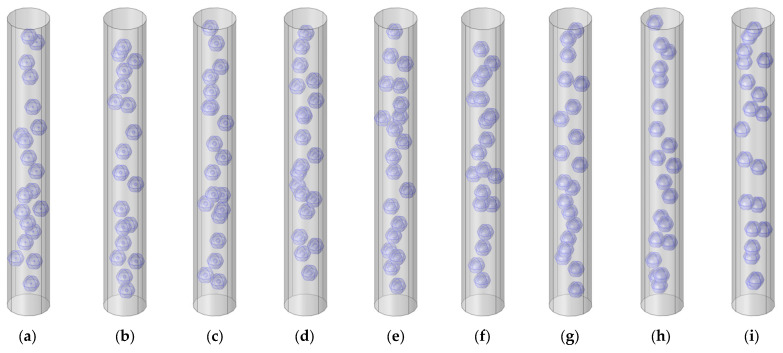
Acoustic finite element simulation models of the composite rubber reinforced with the HGM for the various hollow ratio of the HGM. (**a**) 10%; (**b**) 20%; (**c**) 30%; (**d**) 40%; (**e**) 50%; (**f**) 60%; (**g**) 70%; (**h**) 80%; (**i**) 90%.

Similarly, the finite element simulation models of the composite rubber reinforced with HGM for various hollow ratios of the HGM μ_m_ were constructed, as shown in Figure 10. The selected μ_h_ was in the range of 10% to 90% with an interval of 10%. Based on the built finite element simulation models and the selected parameters, the STL data with the various hollow ratios of the HGM μ_h_ were achieved, as shown in Table 5. It could be found that the STL data at each frequency point in the range of 100–3150 Hz decreased normally along with the increase in the hollow ratio of the HGM, both in the low and high frequency ranges.

**Figure 11:**
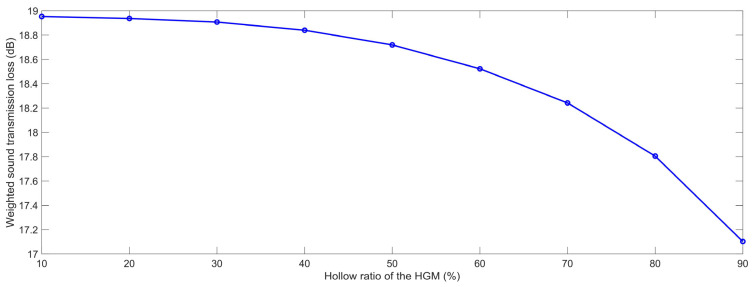
The weighted STL of the composite rubber reinforced with HGM for the various hollow ratio of the HGM.

The variation of the weighted STL along with the increase in the hollow ratio of the HGM μ_h_ is shown in Figure 11. It could be found that the relationship between the weighted STL and the hollow ratio of the HGM was negative, particularly when the value of the μ_h_ was larger than 50%. Along with the increase in the hollow ratio, from 10% to 90%, the weighted STL data decreased from 18.95 dB to 17.10 dB. The major reason for this phenomenon was that with the increase in the hollow ratio of the HGM μ_h_, the thickness of the wall of the HGM reduced gradually, as shown in the acoustic wave transmission process in the composite rubber reinforced with HGM in Figure 3, and its influence on the sound insulation performance was larger than that of the increase in the interface between the HGM and the air inside it, which would result in a decrease in the STL. The variation of the weighted STL was remarkable, which indicated that the hollow ratio of the HGM μ_h_ was an important controllable factor for adjusting the sound insulation performance of the composite rubber reinforced with HGM. Meanwhile, the small hollow ratio indicated the reduction in the cavity in the HGM, which would increase the actual weight of the HGM. Therefore, the appropriate hollow ratio should be established to give consideration to both the sound insulation performance and the weight of the sound insulator.

#### 3.1.4. Thickness of the Sound Insulator

**Figure 12:**
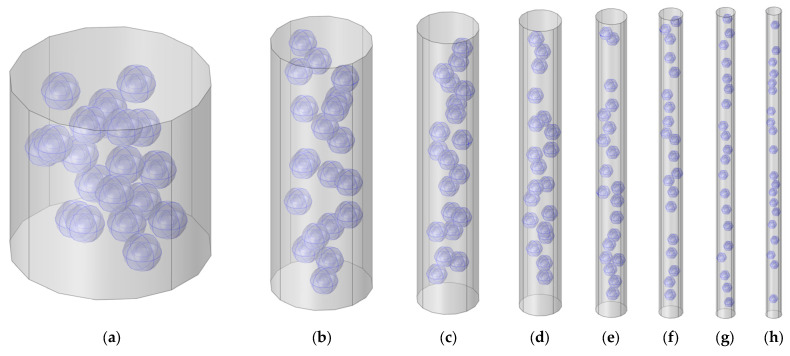
Acoustic finite element simulation models of composite rubber reinforced with HGM for various thickness of the sound insulator. (**a**) 0.5 mm; (**b**) 1.0 mm; (**c**) 1.5 mm; (**d**) 2.0 mm; (**e**) 2.5 mm; (**f**) 3.0 mm; (**g**) 3.5 mm; (**h**) 4.0 mm.

In the same way, the acoustic finite element simulation models of the composite rubber reinforced with HGM for various thicknesses of the sound insulator T were constructed, as shown in the Figure 12. The selected T was in the range of 0.5 mm to 4.0 mm, with an interval of 0.5 mm. With the increase in the thickness T, the distribution of the HGMs were more decentralized in the composite rubber, as shown in the Figure 12. According to the built finite element simulation models and the selected parameters, the STL data with the various thicknesses of the sound insulator T were achieved, which are summarized in Table 6. It could be observed that the STL was significantly affected by the thickness T in the low frequency range, the middle frequency area or in the high frequency region.

**Figure 13:**
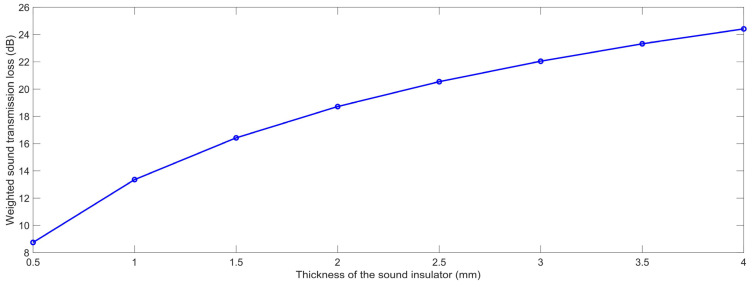
The weighted STL of the composite rubber reinforced with HGM for various thickness of the sound insulator.

The variation of the weighted STL along with the increase in the thickness of the sound insulator T is shown in Figure 13. It could be observed that the relationship between the weighted STL and the thickness of the sound insulator was positive, which was consistent with the normal sound insulation principle of viscoelastic materials. As shown in the acoustic wave transmission process in the composite rubber reinforced with HGM in Figure 3, the consumption of the incident sound wave increased along with the thickness of the sound insulator, because the propagation length of the sound wave increased, and the number of the interfaces raised simultaneously. Meanwhile, it could be found that the improvement in the weighted STL slowed down with the continuous increase in the thickness. Therefore, the thickness T should be confirmed to give consideration to both the sound insulation performance and the occupied space.

### 3.2. Physical Parameters

In addition to these structural parameters, four physical parameters were investigated in this study, which included the density of the composite rubber ρ_r_, the density of the HGM ρ_m_, the acoustic velocity in the composite rubber C_r_ and the acoustic velocity in the HGM C_h_. These four physical parameters were studied successively in this section. In contrast to the analysis of the structural parameters, the analysis of the physical parameters does not require a new acoustic finite element simulation model to be built because the alteration of the physical parameters had no influence on the three–dimensional structures of the model. Therefore, the acoustic finite element simulation model in Figure 1 was utilized, and the influence of the physical parameters was analyzed by changing the values of the corresponding parameters.

#### 3.2.1. Density of the Composite Rubber

**Figure 14:**
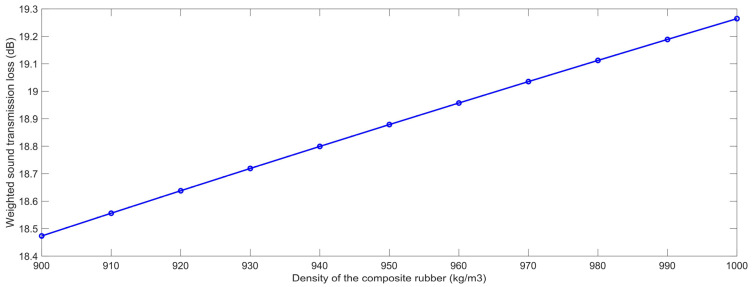
The weighted STL of composite rubber reinforced with HGM for the various density of the composite rubber.

Similarly, The STL data with the various densities of the composite rubber ρ_r_ were gained, which are shown in Table 7. The variation of the weighted STL along with the increase in the ρ_r_ is shown in Figure 14. It could be found that the relationship between the weighted STL and ρ_r_ was positive. The major reason for this phenomenon was that the sound insulation performance of the composite rubber reinforced with HGM obeyed the law of quality control, which meant that the weighted STL could increase along with the density of the composite rubber ρ_r_. However, the improvement of the weighted STL was smaller than 0.8 dB when the value of the ρ_r_ increased from 900 kg/m^3^ to 1000 kg/m^3^, because the actual variable range for the density of the composite rubber ρ_r_ was limited, which meant that the ρ_r_ had little impact on the sound insulation effect of the sound insulator as well.

#### 3.2.2. Density of the HGM

**Figure 15:**
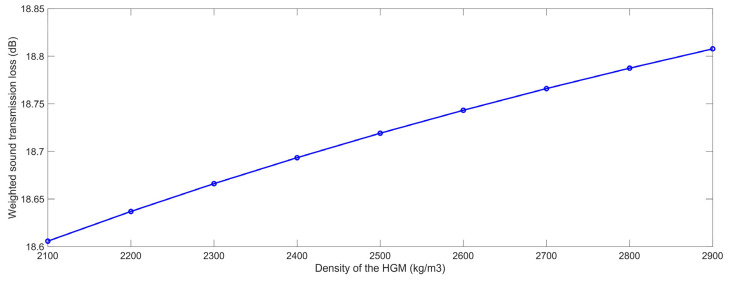
The weighted STL of composite rubber reinforced with HGM for the various density of the HGM.

Similarly, according to the constructed finite element simulation models in Figure 1 and the selected parameters in Table 1, the STL data with the various densities of the HGM ρ_m_ were gained, which are summarized in Table 8, and the investigated values were in the range of 2100 kg/m^3^ to 2900 kg/m^3^ with an interval of 100 kg/m^3^. The variation of the weighted STL along with the increase in the ρ_m_ is shown in Figure 15. It could be found that the relationship between the weighted STL and ρ_m_ was also positive. Similarly, the major reason for this phenomenon was that the sound insulation performance of the composite rubber reinforced with HGM obeyed the law of quality control, which meant that the weighted STL could increase along with the density of the HGM ρ_m_. However, as shown in the acoustic wave transmission process in the composite rubber reinforced with HGM in Figure 3, the increase in the density of the HGM ρ_m_ had little influence on the propagation length of the sound wave and the number of interfaces; therefore, the improvement of the weighted STL was near 0.2 dB when the value of the ρ_m_ increased from 2100 kg/m^3^ to 2900 kg/m^3^, which indicated that the density of the HGM ρ_m_ also had little impact on the sound insulation effect of the sound insulator.

#### 3.2.3. Acoustic Velocity in the Composite Rubber

**Figure 16:**
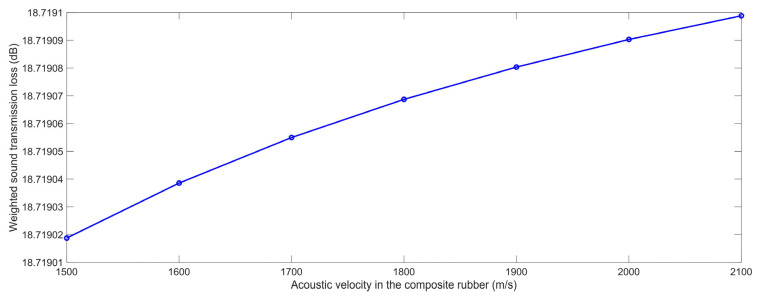
The weighted STL of composite rubber reinforced with HGM for the various acoustic velocity in the composite rubber.

In the same way, according to the constructed finite element simulation models in Figure 1 and the selected parameters in Table 1, the STL data with the various acoustic velocities in the composite rubber C_r_ for the range of 1500 m/s to 2100 m/s with the interval of 100 m/s were obtained, which are shown in Table 9, and the variation of the weighted STL along with the increase in the C_r_ is shown in Figure 16. Although the relationship between the weighted STL and the C_r_ was positive judging from Figure 16, the actual weighted STL had almost no change and the variation was smaller than 0.0001 dB, judging from Table 9, which indicates that the C_r_ should not be of concern in the development of a sound insulator using composite rubber reinforced with HGM. The major reason for this phenomenon was that the acoustic velocity in the composite rubber C_r_ was determined by the characteristic parameters of the rubber, such as density, rigidity, hardness, etc.; therefore, the increase in the C_r_ itself would not affect the sound insulation performance. In fact, the acoustic velocity in the composite rubber C_r_ was difficult to control and to detect for the actual composite rubber, it was only an investigated parameter in the acoustic finite element model as an influencing factor.

#### 3.2.4. Acoustic Velocity in the HGM

**Figure 17:**
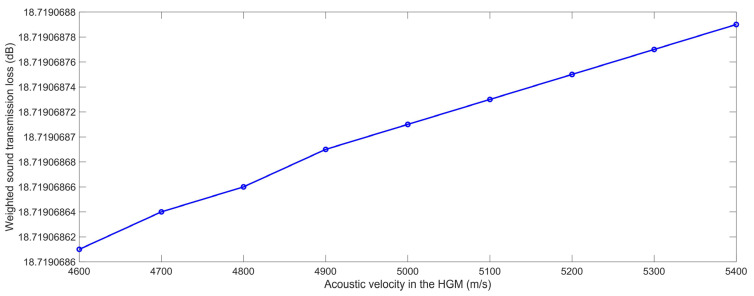
The weighted STL of the composite rubber reinforced with HGM for the various acoustic velocity in the HGM.

In the same light, according to the constructed finite element simulation models in Figure 1 and the selected parameters in Table 1, the STL data with the various acoustic velocities in the HGM C_m_ were achieved for the range of 4600 m/s to 5400 m/s, with an interval of 100 m/s, which are summarized in Table 10, and the variation of the weighted STL along with the increase in the C_m_ is shown in Figure 17. Similarly, it could be judged from Figure 16 that the relationship between the weighted STL and the C_m_ was positive, and the actual weighted STL had almost no change and the variation was smaller than 0.000002 dB, judging from Table 10. The major reason for this phenomenon was similar to the analysis in Section 3.2.3 for the acoustic velocity in the composite rubber C_r_.

Therefore, it could be concluded that the sound insulation performance was insensitive to the density of the composite rubber and that of the HGM, or the acoustic velocity in the composite rubber and that in the HGM, which indicated that the selections of the type of composite rubber and HGM had almost no influence on the sound insulation performance. This feature was favorable for developing various kinds of sound insulators made up of composite rubber reinforced with HGM for different practical applications by using the suitable rubber (such as butadiene styrene rubber, polyisoprene rubber, fluororubber, butyl rubber, polyurethane rubber, polybutadiene rubber, nitrile rubber, silicon rubber, ethylene propylene rubber, etc.) and the appropriate HGM, which could obtain fine thermal, mechanical, electrical or other properties, as desired.

## 4. Results and Discussions

It could be judged from the analysis of these influencing parameters that the thickness was the most important factor for determining the sound insulation performance of the composite rubber reinforced with HGM, and the structural parameters (diameter, volume ratio, and hollow ratio of the HGM and thickness of the sound insulator) had a larger influence on the sound insulation property than the physical parameters (the density of composite rubber and that of HGM, and the acoustic velocity in the composite rubber and that in the HGM).

In order to exhibit the effect of the filled HGM, the sound insulation performance of the pure composite rubber without any reinforcement was analyzed through the acoustic finite element simulation, which could be treated as the contrast. Afterward, the sound insulation performance of the composite rubber reinforced with HGM with different thicknesses was improved through selecting the suitable influencing parameters.

### 4.1. Sound Insulation Performance of Pure Composite Rubber

**Figure 18:**
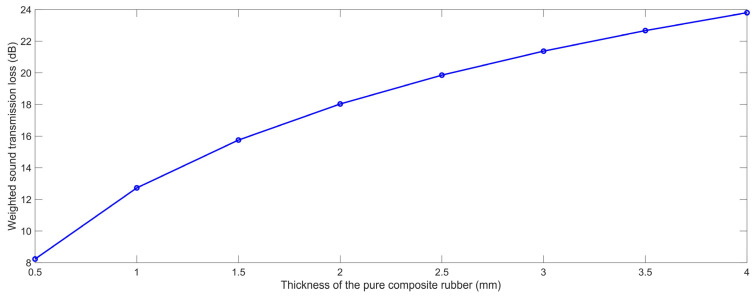
The weighted STL of the pure composite rubber with the various thickness.

According to the built finite element simulation model in Figure 1, without the generation of the HGM, and the reference values of these parameters in Table 1, the STL data with the various thicknesses of the sound insulator T were achieved, which are summarized in Table 11 and shown in Figure 18, and the investigated values were in the range of 0.5 mm to 4.0 mm with an interval of 0.5 mm. Its characteristic was consistent with the normal sound insulation principle of viscoelastic materials. In the majority of cases, the sound insulation performance of the viscoelastic material was determined by its mass. In other words, it was determined by the thickness when the density was kept constant.

Meanwhile, it could be found that the increased range descended along with the increase in the frequency. Taking the sound insulator with the thickness of 0.5 mm and that with the thickness of 1.0 mm, for example, the increase ranges were 247.30%, 227.33%, 201.12%, 175.33%, 149.63%, 125.02%, 103.08%, 86.17%, 72.10%, 60.68%, 52.30%, 45.63%, 39.77%, 35.52%, 32.02% and 29.02%, corresponding to frequencies 100 Hz, 125 Hz, 160 Hz, 200 Hz, 250 Hz, 315 Hz, 400 Hz, 500 Hz, 630 Hz, 800 Hz, 1000 Hz, 1250 Hz, 1600 Hz, 2000 Hz, 2500 Hz and 3150 Hz, respectively. The major reason for this phenomenon was that the sound wave with a higher frequency was easier to be reflected by the interface between the ambient air and rubber, as shown in the acoustic wave transmission process in the composite rubber in Figure 3. This meant a larger reflected sound energy E_Reflection_ and a smaller actual incident sound energy. By contrast, the sound wave with a lower frequency had a stronger penetration capacity, which could penetrate into the composite rubber better and be consumed more thoroughly with the increase in the thickness.

Moreover, for a certain frequency, the increased range descended along with the increase in the thickness T. Taking the frequency 200 Hz, for example, the increase ranges were 175.33%, 56.66%, 29.62%, 18.89%, 13.42%, 10.20% and 8.12% corresponding to thicknesses 1.0 mm, 1.5 mm, 2.0 mm, 2.5 mm, 3.0 mm, 3.5 mm and 4.0 mm, respectively, because the sound insulation performance was not completely linear to the thickness and its influence decreased with the continuous increase in the thickness. Furthermore, the weighted STL of the pure composite rubber reached 12.72 dB, 18.03 dB, 21.37 dB and 23.80 dB with the thickness of 1.0 mm, 2.0 mm, 3.0 mm and 4.0 mm, respectively, which exhibited an excellent sound insulation performance and took little occupied space. The results were basically consistent with the experimental data expressed in the literatures [52,53,54].

### 4.2. Comparative Analysis

**Figure 19:**
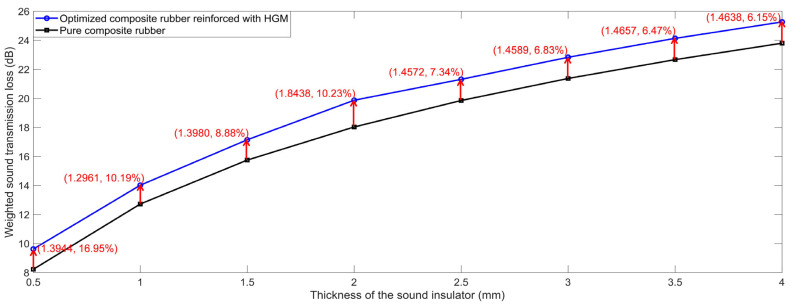
Comparisons of sound insulation performance of pure composite rubber and that of the optimized composite rubber reinforced with HGM.

According to the analysis results of the influencing parameters on the sound insulation performance, and taking into consideration the common optional material for practical application as well, the selected parameters of the optimal composite rubber reinforced with HGM were D_m_ = 20 μm, μ_v_ = 17%, μ_h_ = 10%, ρ_r_ = 1000 kg/m^3^, ρ_m_ = 2900 kg/m^3^, C_r_ = 2100 m/s and C_m_ = 5400 m/s, and the investigated T ranged between 0.5 mm to 4.0 mm, with an interval of 0.5 mm. The STL data with the various thicknesses of the sound insulator T were summarized in Table 12, and the comparisons of the sound insulation performance of the pure composite rubber and that of the optimized composite rubber reinforced with HGM are shown in Figure 19. It could be found that the sound insulation performance was effectively improved through the reinforcement with HGM. In particular, when the T was 0.5 mm, the weighted STL rose from 8.23 dB to 9.62 dB, and the increase range reached 16.95%. The improved sound insulation performance would promote the application of the proposed sound insulator.

## 5. Conclusions

According to the constructed acoustic finite element simulation model based on the basic theory of pressure acoustics, the sound insulation performance and mechanism of the composite rubber reinforced with HGM for various influencing parameters was analyzed in this research, and the major achievements were as follows.

(1) Through the analysis of the sound insulation mechanism with the distribution of SP and SPL in the built acoustic finite element simulation models, it could be concluded that the sound insulation effect of the composite rubber reinforced with HGM was realized through: the reflection and diffraction of the sound wave at the interfaces; the extension of the transmission path of sound wave; the reflection of the sound wave in the hollow structure; and the expansion and compression of the air in the cavity. The exhibited sound insulation mechanism would explain the different sound insulation performances with various parameters for the composite rubber reinforced with HGM.
(2) There were four structural parameters and four physical parameters investigated, and the weighted STL with 1/3 octave band was selected as the evaluating indicator. It could be concluded that the diameter D_m_, volume ratio μ_v_ and hollow ratio μ_h_ of the HGM had a negative effect on the sound insulation performance, and the other five parameters (T, ρ_r_, ρ_m_, C_r_ and C_m_) had a positive effect. Meanwhile, the thickness T was the most influential parameter, and the influences of the C_r_ and C_m_ were negligible within the given value range. These summarized characteristics for the various influencing parameters would provide effective guidance for the selection of parameters and the development of various sound insulation materials for different application requirements.
(3) The weighted STL of the optimized composite rubber reinforced with HGM was up to 14.02 dB, 19.88 dB, 22.83 dB and 25.27 dB, with the limited thickness of 1 mm, 2 mm, 3 mm and 4 mm, respectively, which obtained the increase ranges of 10.19%, 10.23%, 6.83% and 6.15%, relative to the composite rubber without any reinforcement. The improvement would not only promote the application of the proposed sound insulator of the composite rubber reinforced with HGM, but also provide a reference for the development of other sound insulation materials.

## Figures and Tables

**Table 1:** Summary of value ranges of the parameters in acoustic finite element simulation model.

Parameters	Symbol	Unit	Value Ranges	Reference Value
Acoustic velocity in the composite rubber	C_r_	m/s	1500–2100	1800
Density of the composite rubber	ρ_r_	Kg/m^3^	900–1000	950
Acoustic velocity in the HGM	C_m_	m/s	4600–5400	5000
Density of the HGM	ρ_m_	Kg/m^3^	2100–2900	2500
Diameter of the HGM	D_m_	μm	20–500	100
Volume ratio of the HGM	μ_v_	%	2.5–17	10
Hollow ratio of the HGM	μ_h_	%	10–90	50
Thickness of the sound insulator	T	mm	0.5–4	2

**Table 2:** The summarized STL data with the various calculation quantity of the HGM.

	5	10	20	30	40	50
100 Hz	5.1159	5.0825	5.0754	5.0768	5.0753	5.0729
125 Hz	6.5438	6.5063	6.4982	6.4998	6.4981	6.4954
160 Hz	8.2956	8.2545	8.2456	8.2474	8.2456	8.2426
200 Hz	9.9956	9.9522	9.9429	9.9447	9.9428	9.9396
250 Hz	11.7740	11.7290	11.7193	11.7212	11.7192	11.7159
315 Hz	13.6724	13.6264	13.6164	13.6183	13.6163	13.6129
400 Hz	15.6747	15.6280	15.6178	15.6198	15.6177	15.6143
500 Hz	17.5685	17.5215	17.5111	17.5131	17.5109	17.5076
630 Hz	19.5445	19.4974	19.4870	19.4889	19.4866	19.4834
800 Hz	21.5959	21.5490	21.5384	21.5403	21.5378	21.5349
1000 Hz	23.5155	23.4691	23.4583	23.4601	23.4574	23.4548
1250 Hz	25.4346	25.3890	25.3780	25.3796	25.3767	25.3746
1600 Hz	27.5524	27.5083	27.4969	27.4982	27.4948	27.4936
2000 Hz	29.4566	29.4149	29.4029	29.4037	29.3995	29.3997
2500 Hz	31.3441	31.3061	31.2931	31.2932	31.2879	31.2902
3150 Hz	33.2700	33.2383	33.2237	33.2226	33.2154	33.2213
Weighted STL	18.7721	18.7295	18.7191	18.7205	18.7176	18.7159

**Table 3:** The summarized STL data with the various diameter of the HGM.

	20 μm	30 μm	40 μm	50 μm	60 μm	80 μm	100 μm	150 μm	200 μm	300 μm	500 μm
100 Hz	5.1398	5.1017	5.0883	5.0830	5.0734	5.0702	5.0754	5.0729	5.0742	5.0740	5.0720
125 Hz	6.5707	6.5279	6.5127	6.5067	6.4960	6.4923	6.4982	6.4954	6.4969	6.4967	6.4944
160 Hz	8.3250	8.2781	8.2616	8.2550	8.2432	8.2392	8.2456	8.2426	8.2442	8.2440	8.2414
200 Hz	10.0268	9.9772	9.9597	9.9527	9.9403	9.9361	9.9429	9.9397	9.9413	9.9411	9.9384
250 Hz	11.8063	11.7548	11.7367	11.7295	11.7166	11.7123	11.7193	11.7159	11.7177	11.7174	11.7146
315 Hz	13.7056	13.6528	13.6343	13.6269	13.6136	13.6092	13.6164	13.6129	13.6147	13.6144	13.6115
400 Hz	15.7086	15.6547	15.6361	15.6285	15.6150	15.6106	15.6178	15.6143	15.6161	15.6157	15.6128
500 Hz	17.6028	17.5482	17.5296	17.5219	17.5082	17.5038	17.5111	17.5074	17.5093	17.5088	17.5059
630 Hz	19.5794	19.5239	19.5056	19.4978	19.4839	19.4796	19.4870	19.4831	19.4851	19.4843	19.4814
800 Hz	21.6313	21.5750	21.5571	21.5492	21.5352	21.5311	21.5384	21.5343	21.5363	21.5352	21.5324
1000 Hz	23.5516	23.4942	23.4772	23.4691	23.4549	23.4511	23.4583	23.4538	23.4560	23.4544	23.4516
1250 Hz	25.4717	25.4127	25.3970	25.3886	25.3742	25.3710	25.3780	25.3729	25.3753	25.3729	25.3702
1600 Hz	27.5913	27.5295	27.5161	27.5074	27.4925	27.4902	27.4969	27.4909	27.4935	27.4897	27.4871
2000 Hz	29.4980	29.4323	29.4224	29.4130	29.3976	29.3966	29.4029	29.3953	29.3985	29.3925	29.3900
2500 Hz	31.3895	31.3176	31.3132	31.3028	31.2864	31.2875	31.2931	31.2833	31.2871	31.2777	31.2751
3150 Hz	33.3221	33.2400	33.2446	33.2327	33.2148	33.2193	33.2237	33.2100	33.2149	33.1998	33.1962
Weighted STL	18.8075	18.7513	18.7370	18.7290	18.7154	18.7125	18.7191	18.7141	18.7163	18.7136	18.7109

**Table 4:** The summarized STL data with the various volume ratio of the HGM.

	2.50%	5.00%	7.50%	10.00%	12.50%	15.00%	17.00%
100 Hz	4.8259	4.9070	4.9906	5.0754	5.1607	5.2475	5.3191
125 Hz	6.2163	6.3082	6.4026	6.4982	6.5941	6.6915	6.7716
160 Hz	7.9360	8.0371	8.1409	8.2456	8.3506	8.4569	8.5441
200 Hz	9.6150	9.7223	9.8322	9.9429	10.0536	10.1657	10.2575
250 Hz	11.3788	11.4904	11.6044	11.7193	11.8340	11.9500	12.0450
315 Hz	13.2674	13.3818	13.4988	13.6164	13.7338	13.8525	13.9496
400 Hz	15.2637	15.3798	15.4986	15.6178	15.7368	15.8570	15.9553
500 Hz	17.1546	17.2716	17.3913	17.5111	17.6306	17.7515	17.8503
630 Hz	19.1302	19.2473	19.3672	19.4870	19.6062	19.7270	19.8257
800 Hz	21.1837	21.3002	21.4196	21.5384	21.6565	21.7764	21.8743
1000 Hz	23.1080	23.2232	23.3414	23.4583	23.5741	23.6924	23.7887
1250 Hz	25.0356	25.1482	25.2644	25.3780	25.4901	25.6053	25.6990
1600 Hz	27.1691	27.2771	27.3893	27.4969	27.6021	27.7118	27.8006
2000 Hz	29.0965	29.1978	29.3041	29.4029	29.4979	29.5995	29.6811
2500 Hz	31.0207	31.1113	31.2084	31.2931	31.3722	31.4610	31.5313
3150 Hz	33.0070	33.0800	33.1619	33.2237	33.2765	33.3441	33.3957
Weighted STL	18.4005	18.5052	18.6135	18.7191	18.8231	18.9306	19.0181

**Table 5:** The summarized STL data with the various hollow ratio of the HGM.

	10%	20%	30%	40%	50%	60%	70%	80%	90%
100 Hz	5.2233	5.2125	5.1947	5.1536	5.0754	4.9553	4.7713	4.4861	4.0356
125 Hz	6.6645	6.6524	6.6324	6.5862	6.4982	6.3627	6.1542	5.8285	5.3075
160 Hz	8.4277	8.4145	8.3926	8.3420	8.2456	8.0968	7.8670	7.5057	6.9214
200 Hz	10.1354	10.1214	10.0982	10.0448	9.9429	9.7852	9.5412	9.1561	8.5286
250 Hz	11.9193	11.9048	11.8807	11.8252	11.7193	11.5551	11.3009	10.8986	10.2397
315 Hz	13.8221	13.8072	13.7824	13.7253	13.6164	13.4473	13.1856	12.7708	12.0890
400 Hz	15.8281	15.8129	15.7875	15.7290	15.6178	15.4447	15.1775	14.7534	14.0554
500 Hz	17.7253	17.7098	17.6839	17.6242	17.5111	17.3346	17.0632	16.6325	15.9234
630 Hz	19.7054	19.6897	19.6631	19.6021	19.4870	19.3065	19.0310	18.5943	17.8759
800 Hz	21.7627	21.7466	21.7191	21.6561	21.5384	21.3527	21.0723	20.6288	19.9013
1000 Hz	23.6903	23.6738	23.6450	23.5795	23.4583	23.2654	22.9788	22.5273	21.7897
1250 Hz	25.6217	25.6045	25.5738	25.5043	25.3780	25.1742	24.8788	24.4156	23.6646
1600 Hz	27.7610	27.7426	27.7086	27.6321	27.4969	27.2741	26.9633	26.4805	25.7073
2000 Hz	29.6963	29.6761	29.6373	29.5509	29.4029	29.1527	28.8200	28.3091	27.5043
2500 Hz	31.6322	31.6095	31.5630	31.4611	31.2931	31.0001	30.6328	30.0779	29.2238
3150 Hz	33.6376	33.6106	33.5518	33.4245	33.2237	32.8600	32.4359	31.8080	30.8722
Weighted STL	18.9533	18.9368	18.9071	18.8401	18.7191	18.5230	18.2421	17.8046	17.1025

**Table 6:** The summarized STL data with the various thickness of the sound insulator.

	0.5 mm	1.0 mm	1.5 mm	2.0 mm	2.5 mm	3.0 mm	3.5 mm	4.0 mm
100 Hz	0.5636	1.9154	3.5145	5.0754	6.5016	7.7753	8.9189	9.9488
125 Hz	0.8512	2.7094	4.6941	6.4982	8.0748	9.4442	10.6514	11.7253
160 Hz	1.3185	3.8364	6.2224	8.2456	9.9468	11.3912	12.6469	13.7534
200 Hz	1.9152	5.0747	7.7703	9.9429	11.7233	13.2138	14.4984	15.6240
250 Hz	2.7092	6.4975	9.4388	11.7193	13.5544	15.0758	16.3794	17.5172
315 Hz	3.7564	8.1289	11.2589	13.6164	15.4891	17.0313	18.3475	19.4929
400 Hz	5.0744	9.9420	13.2080	15.6178	17.5153	19.0708	20.3946	21.5440
500 Hz	6.4971	11.7184	15.0697	17.5111	19.4227	20.9855	22.3130	23.4633
630 Hz	8.1285	13.6155	17.0251	19.4870	21.4070	22.9735	24.3020	25.4506
800 Hz	9.9416	15.6170	19.0643	21.5384	23.4620	25.0291	26.3561	27.4995
1000 Hz	11.7179	17.5103	20.9787	23.4583	25.3813	26.9461	28.2687	29.4032
1250 Hz	13.5488	19.4179	22.8975	25.3780	27.2965	28.8556	30.1701	31.2898
1600 Hz	15.6162	21.5378	25.0208	27.4969	29.4046	30.9522	32.2514	33.3443
2000 Hz	17.5094	23.4578	26.9364	29.4029	31.2933	32.8233	34.0999	35.1532
2500 Hz	19.4166	25.3777	28.8438	31.2931	33.1556	34.6575	35.8981	36.8884
3150 Hz	21.4005	27.3620	30.8038	33.2237	35.0396	36.4946	37.6753	38.5602
Weighted STL	8.7478	13.3574	16.4217	18.7191	20.5417	22.0450	23.3232	24.4161

**Table 7:** The summarized STL data with the various density of the composite rubber.

	900 kg/m^3^	910 kg/m^3^	920 kg/m^3^	930 kg/m^3^	940 kg/m^3^	950 kg/m^3^	960 kg/m^3^	970 kg/m^3^	980 kg/m^3^	990 kg/m^3^	1000 kg/m^3^
100 Hz	4.8957	4.9559	5.0158	5.0754	5.1347	5.1937	5.2523	5.3107	5.3688	5.4266	5.4841
125 Hz	6.2953	6.3634	6.4310	6.4982	6.5649	6.6311	6.6969	6.7623	6.8272	6.8917	6.9558
160 Hz	8.0229	8.0978	8.1720	8.2456	8.3187	8.3911	8.4629	8.5342	8.6049	8.6750	8.7445
200 Hz	9.7071	9.7864	9.8650	9.9429	10.0200	10.0965	10.1722	10.2473	10.3217	10.3954	10.4685
250 Hz	11.4743	11.5568	11.6384	11.7193	11.7993	11.8786	11.9570	12.0348	12.1118	12.1880	12.2636
315 Hz	13.3650	13.4497	13.5335	13.6164	13.6984	13.7797	13.8601	13.9396	14.0184	14.0964	14.1737
400 Hz	15.3622	15.4483	15.5335	15.6178	15.7012	15.7838	15.8654	15.9463	16.0263	16.1054	16.1838
500 Hz	17.2529	17.3399	17.4260	17.5111	17.5953	17.6787	17.7611	17.8426	17.9233	18.0032	18.0823
630 Hz	19.2271	19.3147	19.4013	19.4870	19.5717	19.6555	19.7384	19.8205	19.9016	19.9820	20.0614
800 Hz	21.2775	21.3655	21.4524	21.5384	21.6235	21.7076	21.7908	21.8731	21.9545	22.0351	22.1149
1000 Hz	23.1970	23.2851	23.3722	23.4583	23.5435	23.6278	23.7111	23.7935	23.8751	23.9558	24.0356
1250 Hz	25.1165	25.2047	25.2918	25.3780	25.4632	25.5475	25.6308	25.7133	25.7948	25.8755	25.9554
1600 Hz	27.2358	27.3238	27.4109	27.4969	27.5820	27.6662	27.7494	27.8317	27.9132	27.9938	28.0735
2000 Hz	29.1424	29.2302	29.3171	29.4029	29.4877	29.5716	29.6546	29.7367	29.8179	29.8983	29.9777
2500 Hz	31.0340	31.1213	31.2077	31.2931	31.3776	31.4610	31.5436	31.6253	31.7060	31.7859	31.8650
3150 Hz	32.9667	33.0534	33.1391	33.2237	33.3074	33.3902	33.4720	33.5529	33.6330	33.7121	33.7905
Weighted STL	18.4733	18.5561	18.6380	18.7191	18.7993	18.8788	18.9574	19.0353	19.1124	19.1888	19.2644

**Table 8:** The summarized STL data with the various density of the HGM.

	2100 kg/m^3^	2200 kg/m^3^	2300 kg/m^3^	2400 kg/m^3^	2500 kg/m^3^	2600 kg/m^3^	2700 kg/m^3^	2800 kg/m^3^	2900 kg/m^3^
100 Hz	4.9922	5.0150	5.0364	5.0565	5.0754	5.0932	5.1100	5.1259	5.1409
125 Hz	6.4044	6.4302	6.4543	6.4769	6.4982	6.5182	6.5371	6.5550	6.5719
160 Hz	8.1428	8.1711	8.1975	8.2223	8.2456	8.2676	8.2883	8.3078	8.3263
200 Hz	9.8341	9.8640	9.8920	9.9182	9.9429	9.9661	9.9879	10.0086	10.0281
250 Hz	11.6063	11.6374	11.6665	11.6937	11.7193	11.7433	11.7660	11.7874	11.8077
315 Hz	13.5005	13.5324	13.5622	13.5902	13.6164	13.6411	13.6643	13.6863	13.7070
400 Hz	15.5000	15.5324	15.5628	15.5912	15.6178	15.6429	15.6666	15.6889	15.7100
500 Hz	17.3921	17.4249	17.4555	17.4842	17.5111	17.5365	17.5603	17.5829	17.6042
630 Hz	19.3672	19.4002	19.4310	19.4599	19.4870	19.5125	19.5365	19.5592	19.5806
800 Hz	21.4182	21.4513	21.4823	21.5112	21.5384	21.5640	21.5881	21.6109	21.6324
1000 Hz	23.3379	23.3711	23.4021	23.4311	23.4583	23.4840	23.5081	23.5309	23.5524
1250 Hz	25.2575	25.2907	25.3217	25.3508	25.3780	25.4036	25.4278	25.4506	25.4721
1600 Hz	27.3766	27.4097	27.4407	27.4697	27.4969	27.5225	27.5467	27.5694	27.5910
2000 Hz	29.2829	29.3159	29.3468	29.3757	29.4029	29.4284	29.4525	29.4752	29.4966
2500 Hz	31.1737	31.2066	31.2373	31.2661	31.2931	31.3185	31.3425	31.3651	31.3864
3150 Hz	33.1053	33.1379	33.1684	33.1969	33.2237	33.2489	33.2726	33.2950	33.3162
Weighted STL	18.6057	18.6369	18.6661	18.6934	18.7191	18.7432	18.7660	18.7874	18.8077

**Table 9:** The summarized STL data with the various acoustic velocity in the composite rubber.

	1500 m/s	1600 m/s	1700 m/s	1800 m/s	1900 m/s	2000 m/s	2100 m/s
100 Hz	5.0754	5.0754	5.0754	5.0754	5.0754	5.0754	5.0754
125 Hz	6.4982	6.4982	6.4982	6.4982	6.4982	6.4982	6.4982
160 Hz	8.2456	8.2456	8.2456	8.2456	8.2456	8.2456	8.2456
200 Hz	9.9429	9.9429	9.9429	9.9429	9.9429	9.9429	9.9429
250 Hz	11.7193	11.7193	11.7193	11.7193	11.7193	11.7193	11.7193
315 Hz	13.6164	13.6164	13.6164	13.6164	13.6164	13.6164	13.6164
400 Hz	15.6178	15.6178	15.6178	15.6178	15.6178	15.6178	15.6178
500 Hz	17.5111	17.5111	17.5111	17.5111	17.5111	17.5111	17.5111
630 Hz	19.4870	19.4870	19.4870	19.4870	19.4870	19.4870	19.4870
800 Hz	21.5384	21.5384	21.5384	21.5384	21.5384	21.5384	21.5384
1000 Hz	23.4583	23.4583	23.4583	23.4583	23.4583	23.4583	23.4583
1250 Hz	25.3780	25.3780	25.3780	25.3780	25.3780	25.3780	25.3780
1600 Hz	27.4969	27.4969	27.4969	27.4969	27.4969	27.4970	27.4970
2000 Hz	29.4028	29.4028	29.4028	29.4029	29.4029	29.4029	29.4029
2500 Hz	31.2929	31.2930	31.2931	31.2931	31.2932	31.2932	31.2932
3150 Hz	33.2234	33.2235	33.2236	33.2237	33.2238	33.2238	33.2239
Weighted STL	18.7190	18.7190	18.7191	18.7191	18.7191	18.7191	18.7191

**Table 10:** The summarized STL data with the various acoustic velocity in the HGM.

	4600 m/s	4700 m/s	4800 m/s	4900 m/s	5000 m/s	5100 m/s	5200 m/s	5300 m/s	5400 m/s
100 Hz	5.0754	5.0754	5.0754	5.0754	5.0754	5.0754	5.0754	5.0754	5.0754
125 Hz	6.4982	6.4982	6.4982	6.4982	6.4982	6.4982	6.4982	6.4982	6.4982
160 Hz	8.2456	8.2456	8.2456	8.2456	8.2456	8.2456	8.2456	8.2456	8.2456
200 Hz	9.9429	9.9429	9.9429	9.9429	9.9429	9.9429	9.9429	9.9429	9.9429
250 Hz	11.7193	11.7193	11.7193	11.7193	11.7193	11.7193	11.7193	11.7193	11.7193
315 Hz	13.6164	13.6164	13.6164	13.6164	13.6164	13.6164	13.6164	13.6164	13.6164
400 Hz	15.6178	15.6178	15.6178	15.6178	15.6178	15.6178	15.6178	15.6178	15.6178
500 Hz	17.5111	17.5111	17.5111	17.5111	17.5111	17.5111	17.5111	17.5111	17.5111
630 Hz	19.4870	19.4870	19.4870	19.4870	19.4870	19.4870	19.4870	19.4870	19.4870
800 Hz	21.5384	21.5384	21.5384	21.5384	21.5384	21.5384	21.5384	21.5384	21.5384
1000 Hz	23.4583	23.4583	23.4583	23.4583	23.4583	23.4583	23.4583	23.4583	23.4583
1250 Hz	25.3780	25.3780	25.3780	25.3780	25.3780	25.3780	25.3780	25.3780	25.3780
1600 Hz	27.4969	27.4969	27.4969	27.4969	27.4969	27.4969	27.4969	27.4969	27.4969
2000 Hz	29.4029	29.4029	29.4029	29.4029	29.4029	29.4029	29.4029	29.4029	29.4029
2500 Hz	31.2931	31.2931	31.2931	31.2931	31.2931	31.2931	31.2931	31.2931	31.2931
3150 Hz	33.2237	33.2237	33.2237	33.2237	33.2237	33.2237	33.2237	33.2237	33.2237
Weighted STL	18.7191	18.7191	18.7191	18.7191	18.7191	18.7191	18.7191	18.7191	18.7191

**Table 11:** The summarized STL data with the various thickness for the pure composite rubber.

	0.5 mm	1.0 mm	1.5 mm	2.0 mm	2.5 mm	3.0 mm	3.5 mm	4.0 mm
100 Hz	0.4767	1.6556	3.1053	4.5582	5.9114	7.1407	8.2512	9.2561
125 Hz	0.7235	2.3682	4.2022	5.9113	7.4290	8.7659	9.9490	11.0046
160 Hz	1.1293	3.4006	5.6504	7.5985	9.2560	10.6793	11.9185	13.0124
200 Hz	1.6555	4.5581	7.1407	9.2560	11.0046	12.4816	13.7550	14.8718
250 Hz	2.3680	5.9112	8.7658	11.0046	12.8169	14.3306	15.6269	16.7588
315 Hz	3.3266	7.4856	10.5542	12.8825	14.7395	16.2783	17.5900	18.7321
400 Hz	4.5577	9.2558	12.4816	14.8717	16.7587	18.3143	19.6362	20.7849
500 Hz	5.9108	11.0044	14.3305	16.7587	18.6638	20.2293	21.5573	22.7100
630 Hz	7.4852	12.8823	16.2782	18.7320	20.6493	22.2215	23.5535	24.7088
800 Hz	9.2554	14.8715	18.3142	20.7849	22.7100	24.2865	25.6211	26.7781
1000 Hz	11.0039	16.7585	20.2293	22.7100	24.6398	26.2188	27.5550	28.7130
1250 Hz	12.8162	18.6636	22.1526	24.6398	26.5725	28.1532	29.4903	30.6489
1600 Hz	14.8711	20.7847	24.2864	26.7781	28.7129	30.2946	31.6324	32.7914
2000 Hz	16.7580	22.7098	26.2187	28.7129	30.6489	32.2312	33.5692	34.7283
2500 Hz	18.6631	24.6396	28.1531	30.6488	32.5855	34.1681	35.5062	36.6654
3150 Hz	20.6486	26.6414	30.1579	32.6546	34.5916	36.1743	37.5124	38.6714
Weighted STL	8.2281	12.7244	15.7513	18.0314	19.8556	21.3730	22.6703	23.8023

**Table 12:** The summarized STL data of the optimized composite rubber reinforced with HGM for the various thickness.

	0.5 mm	1.0 mm	1.5 mm	2.0 mm	2.5 mm	3.0 mm	3.5 mm	4.0 mm
100 Hz	0.7351	2.2091	3.9688	5.9529	7.1160	8.4298	9.6133	10.6657
125 Hz	1.0997	3.0879	5.2299	7.4747	8.7392	10.1375	11.3770	12.4674
160 Hz	1.6795	4.3096	6.8343	9.3049	10.6509	12.1143	13.3958	14.5142
200 Hz	2.3997	5.6256	8.4356	11.0554	12.4522	13.9550	15.2614	16.3961
250 Hz	3.3297	7.1131	10.1436	12.8688	14.3002	15.8293	17.1519	18.2973
315 Hz	4.5177	8.7961	11.9924	14.7917	16.2468	17.7936	19.1269	20.2792
400 Hz	5.9667	10.6476	13.9615	16.8104	18.2814	19.8396	21.1795	22.3359
500 Hz	7.4899	12.4488	15.8359	18.7141	20.1946	21.7592	23.1025	24.2609
630 Hz	9.2007	14.3633	17.8003	20.6969	22.1840	23.7522	25.0967	26.2555
800 Hz	11.0724	16.3768	19.8463	22.7530	24.2445	25.8141	27.1576	28.3155
1000 Hz	12.8862	18.2778	21.7660	24.6757	26.1702	27.7391	29.0796	30.2349
1250 Hz	14.7425	20.1910	23.6902	26.5973	28.0943	29.6605	30.9952	32.1457
1600 Hz	16.8281	22.3161	25.8209	28.7177	30.2175	31.7776	33.1014	34.2430
2000 Hz	18.7317	24.2409	27.7460	30.6248	32.1277	33.6782	34.9857	36.1141
2500 Hz	20.6457	26.1665	29.6673	32.5163	34.0234	35.5585	36.8402	37.9476
3150 Hz	22.6341	28.1592	31.6498	34.4485	35.9621	37.4717	38.7105	39.7833
Weighted STL	9.6225	14.0206	17.1493	19.8752	21.3128	22.8319	24.1360	25.2660

## Data Availability

The data that support the findings of this study are available from the corresponding author upon reasonable request.
